# Response to Deep Brain Stimulation in Three Brain Targets with Implications in Mental Disorders: A PET Study in Rats

**DOI:** 10.1371/journal.pone.0168689

**Published:** 2016-12-29

**Authors:** Marta Casquero-Veiga, Ravit Hadar, Javier Pascau, Christine Winter, Manuel Desco, María Luisa Soto-Montenegro

**Affiliations:** 1 CIBER de Salud Mental (CIBERSAM), Madrid, Spain; 2 Instituto de Investigación Sanitaria Gregorio Marañón, Madrid, Spain; 3 Department of Psychiatry and Psychotherapy, Medical Faculty Carl Gustav Carus, Technische Universität Dresden, Dresden, Germany; 4 Departamento de Bioingeniería e Ingeniería Aeroespacial, Universidad Carlos III de Madrid, Spain; National Institue on Drug Abuse, UNITED STATES

## Abstract

**Objective:**

To investigate metabolic changes in brain networks by deep brain stimulation (DBS) of the medial prefrontal cortex (mPFC), nucleus accumbens (NAcc) and dorsomedial thalamus (DM) using positron emission tomography (PET) in naïve rats.

**Methods:**

43 male Wistar rats underwent stereotactic surgery and concentric bipolar platinum-iridium electrodes were bilaterally implanted into one of the three brain sites. [^18^F]-fluoro-2-deoxy-glucose-PET (^18^FDG-PET) and computed tomography (CT) scans were performed at the 7th (without DBS) and 9th day (with DBS) after surgery. Stimulation period matched tracer uptake period. Images were acquired with a small-animal PET-CT scanner. Differences in glucose uptake between groups were assessed with Statistical Parametric Mapping.

**Results:**

DBS induced site-specific metabolic changes, although a common increased metabolic activity in the piriform cortex was found for the three brain targets. mPFC-DBS increased metabolic activity in the striatum, temporal and amygdala, and reduced it in the cerebellum, brainstem (BS) and periaqueductal gray matter (PAG). NAcc-DBS increased metabolic activity in the subiculum and olfactory bulb, and decreased it in the BS, PAG, septum and hypothalamus. DM-DBS increased metabolic activity in the striatum, NAcc and thalamus and decreased it in the temporal and cingulate cortex.

**Conclusions:**

DBS induced significant changes in ^18^FDG uptake in brain regions associated with the basal ganglia-thalamo-cortical circuitry. Stimulation of mPFC, NAcc and DM induced different patterns of ^18^FDG uptake despite interacting with the same circuitries. This may have important implications to DBS research suggesting individualized target selection according to specific neural modulatory requirements.

## Introduction

Mental disorders are the third leading cause of disability-adjusted life years (DALYs) loss and the first cause of years lived with disability (YLD) in Europe, accounting for 36.1% of those attributable to all causes[1]. Mental disorders greatly influence patients’ overall health, economic situation and social integration. Even though effective treatment exist, 10–30% of the patients have little or no response to traditional treatment strategies and up to an additional 30% of the patients experience only partial relief [2], thus making it essential to explore other treatments. During the last decades, brain electrical stimulation techniques have emerged in the bio-scientific scenario. Among them, deep brain stimulation (DBS) constitutes a neurosurgery technique that modifies neural activity by means of an electrical current applied directly to specific brain targets. It has been licensed as a treatment option for several movement disorders [3]. The idea to extend DBS to the treatment of psychiatric disorders was based on the notion that psychiatric disorders are the clinical presentation of dysfunctional brain networks and the observation that DBS induces depressive and hypomanic states in Parkinson’s disease patients [4]. Meanwhile, DBS in the ventral capsule/ventral striatum (VC/VS), which contains the nucleus accumbens (NAcc), has received FDA approval for treatment of obsessive compulsive disorders, is being tested for treatment of depressive disorders [5–7] and addiction [8–11] and the first preclinical report on successful DBS in the context of schizophrenia has just been published [12]. The only double-blind sham-controlled trials for chronic treatment-resistant depression stimulated the VC/VS[13] and Brodmann area 25[14], obtaining little success. Thus, it is noteworthy that, with exception of VC/VS-DBS for OCD, there is no much evidence yet supporting open loop DBS for psychiatric indications. Future research applying new study designs and DBS parameters (e.g. close-loop DBS[15]) are needed to confirm its clinical potential. On the other hand, DBS also holds scientific promise in the identification of interconnected functional networks and dysfunctional brain circuits underlying a physiological and pathological brain functions due to its capacity to specifically modify neural discharge patterns locally, at the electrode placement, and remotely, in associated brain areas [16, 17] and affect neural network activity[18–20]. Across the neuro-psychiatric disorders currently subjected to DBS treatment trials, the following DBS targets are being tested: medial prefrontal cortex (mPFC), globus pallidus internus, subthalamic nucleus, zona incerta, nucleus accumbens (NAcc)/ventral striatum, hippocampus and thalamus (centromedian/parafascicularis; anterior nucleus; periaqueductal gray/periventricular gray; ventrolateral intermedius; ventral posterolateral/ventro-posteromedial), lateral habenula, nucleus basali Meynert, medial forebrain bundle (MFB), and fornix/hypothalamus [21–24]. In addition, the mediodorsal thalamic nucleus (DM) structure has been suggested relevant in the context of psychiatric disorders as it interconnects with the dorsolateral PFC and limbic structures, including limbic cortex, hippocampus and basolateral amygdala [25]. Nevertheless, there is no consensus on which area is best for each disorder. Indeed, several areas are being investigated for the same pathology, i.e. mPFC, cingulum, MFB, ventral striatum or the NAcc for depression, STN, NAcc or ventral striatum for obsessive compulsive, mPFC or NAcc for future schizophrenia studies; more to that, some cases, the same area is being investigated for several disorders [26–29]. So far, targets have been selected upon assumptions about the pathophysiological relevance of the respective brain site in the manifestation of the respective disorder but often enough lack a scientific framework that proves the selection. From a theoretical point of view, the optimal DBS target would be the one that mostly interconnects with circuits involved in the manifestation of the symptoms to be targeted.

In this context, functional neuroimaging is a powerful tool in terms of locating brain networks modulated by DBS and refining stimulation protocols [30]. Positron emission tomography (PET) with 2-deoxy-2-[^18^F]fluoro-D-glucose (^18^FDG) constitutes the traditional technique for in vivo direct quantification of regional brain glucose metabolism in humans and rodents [12, 18, 31–35]. The method has proven itself as an excellent tool for promoting our understanding of the neurobiological processes in healthy as well as diseased brains and allows for reliable comparative studies [36–40]. We used here ^18^FDG-PET and statistical parametric mapping (SPM) techniques in rats to compare the metabolic modulation of neural networks by DBS applied to either the mPFC, NAcc or DM, all of which are linked to several known neuropsychiatric disorders [41–44].

## Materials and Methods

### Animals

Forty-three male Wistar rats (275–325 g) were housed in a temperature (24 ± 0.5°C) and humidity controlled *vivarium* with a 12 h light-dark cycle. Commercial rodent laboratory chow and water were available *ad libitum* if not indicated differently. All experimental animal procedures were conducted according to the European Communities Council Directive 2010/63/EU and approved by the Ethics Committee for Animal Experimentation of our hospital (Comité de Ética de Experimentación Animal, CEEA; number ES280790000087).

### Surgery and DBS protocol

Stereotaxic surgeries were carried out on animals anesthetized with a mixture of ketamine (100 mg kg^-1^) and xylazine (10 mg kg^-1^). Concentric bipolar platinum-iridium electrodes (Plastics One, Roanoke, USA) were bilaterally implanted in one of the following targets, according to the Paxinos and Watson rat brain atlas [45]: 1) mPFC; anteroposterior (AP) +3.5 (from Bregma), medio-lateral (ML) +0.6, dorso-ventral (DV) -3.4 (from Dura); 2) NAcc: AP +1.2, ML +1.8, DV -8.1; and 3) DM: AP -2.8, ML +0.75, DV -5.0. Electrodes were fixed to the skull with dental acrylic cement (Technovit^®^). Computed tomography (CT) scans of all the animals were obtained and co-registered to an MRI study of one non-operated animal (anatomical MRI template) to rule out errors in the placement of the electrodes. Only animals with correct electrodes positions were included in the PET study resulting in the following number of animals per group: 1) mPFC: 10, 2) NAcc: 10 and 3) DM: 11.

PET scans were acquired seven and nine days thereafter, preceded by either sham stimulation (baseline-condition) or DBS applied during ^18^FDG-uptake period (DBS-condition) for 45 minutes. DBS was performed with an isolated stimulator (STG1004; Multi Channel Systems GmbH, Reutlingen, Germany) in a constant current mode at 130 Hz and 150 μA with a pulse width of 100 μs. These settings were chosen based on previous studies by our group [18, 19].

### Imaging studies

All animals were scanned using a small-animal PET/CT scanner (ARGUS PET/CT, SEDECAL, Madrid) under anesthesia with isoflurane (3% induction and 1.5% maintenance in 100% O_2_). ^18^FDG (approximately 1 mCi) was injected into the tail vein and, after an uptake period of 45 minutes, animals were scanned for 45 minutes. Images were reconstructed using a 2D-OSEM (ordered subset expectation maximization) algorithm, which claims a spatial resolution for this scanner of 1.45 mm FWHM (full width at half maximum), with a voxel size of 0.3875 x 0.3875 x 0.7750 mm^3^. The energy window was 400–700 keV. Decay and deadtime corrections were applied.

CT studies were acquired with the following parameters: 340 mA, 40 KV, 360 projections, 8 shots per projection, and 200 μm of resolution. CT images were reconstructed using a Feldkamp algorithm (isotropic voxel size of 0.121 mm).

In addition, one MRI scan of a non-operated animal was acquired with a 7-Tesla Biospec 70/20 scanner (Bruker, Ettlingen, Germany) under sevoflurane anesthesia (4.5% for induction and 2.5% for maintenance in 100% O_2,_). A T2-weighted spin echo sequence was acquired, with TE = 33 ms, TR = 3732 ms, and a slice thickness of 0.8 mm (34 slices). The matrix size was 256 × 256 pixels at an FOV of 3.5 × 3.5 cm^2^. This single-animal study was used as an anatomical template in order to display the results of the SPM study.

### Analysis of PET data

CT studies were co-registered to a random reference CT scan using an automatic rigid registration method based on mutual information, and the spatial transformation obtained for each CT image was subsequently applied to the corresponding PET[46]. The single MRI study was also spatially co-registered to the reference CT scan. A brain mask segmented on the MRI study was applied to all registered PET images and the resulting images were smoothed with an isotropic Gaussian filter (2 mm FWHM). Voxel values were normalized to the average white matter intensity in order to obtain the regional characterization of metabolic changes circumventing overall differences in animal brain metabolism. White matter normalization was used in accordance with the criteria of Shinohara et al.[47].

A region of interest (ROI) analysis was performed to determine the global metabolic differences. Whole brain and white matter masks segmented on the MR template were used for this analysis. Whole brain data were normalized to average white matter intensity.

### Statistical analysis

Statistical analysis of regional PET data was performed using the software package SPM12 (Statistical Parametric Mapping, Wellcome Trust Centre for Neuroimaging, London, UK). Groups were compared by means of a paired *t* test with a significance threshold of p<0.01 (T = 2.82), uncorrected for multiple comparisons. To reduce type I error, a 50-voxel clustering threshold (spatial-extent) was applied. Global differences were assessed by means of a paired t-test with a threshold for statistical significance set at p<0.01.

## Results

**Fig 1:**
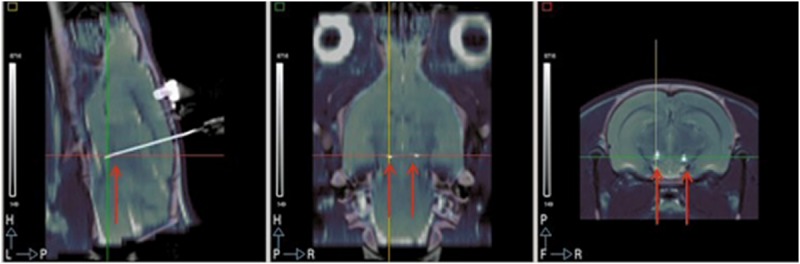
Correct electrode location verification. Sagittal, coronal and axial views of a CT scan registered to the MR template of an animal to verify the correct electrode location. Only animals with electrodes placed correctly in the respective target were included in the study.

Fig 1 shows sagittal, coronal and axial views of a CT scan registered to the MR template of one animal to verify the correct electrode positioning. Only animals with electrodes placed correctly in the respective target were included in the study.

Measurements based on global differences for the whole brain metabolism displayed no significant differences across groups under either treatment, sham-stimulation or DBS. Values for DBS animals were normalized and expressed as a ratio of the average glucose metabolism in the basal time point for each animal: mPFC (0.99±0.020) (p = 0.099), NAcc (1.03±0.15) (p = 0.631) and DM (0.99±0.032) (p = 0.185).

**Fig 2:**
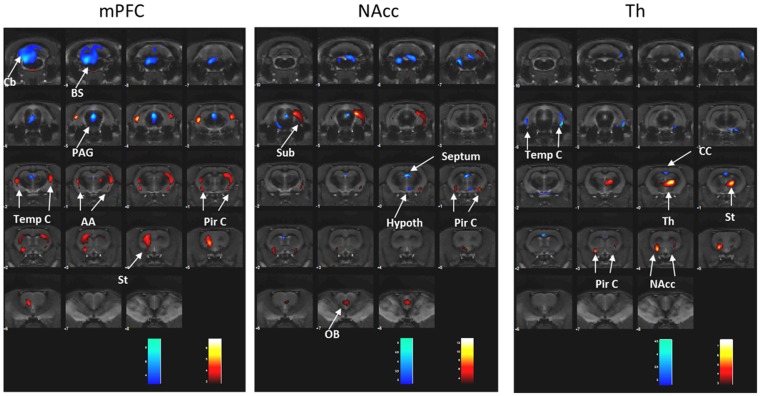
Brain glucose metabolism during DBS in the three brain targets. Effects depend on stimulation target. Colored PET overlays on MR reference indicate increased 18FDG uptake (hot colors) or decreased (cold colors). AA: amygdala; BS: brainstem, Cb: cerebellum, CC: cingulate cortex, Hypoth: hypothalamus, NAcc: nucleus accumbens, PAG: periaqueductal gray matter, Pir C: piriform cortex, Sub: subiculum hippocampal, Str: striatum, Temp C: temporal cortex, Th: thalamus.

mPFC-DBS treatment increased metabolic activity in the striatum, temporal and piriform cortex and amygdala (right: T = 6.39, p<0.001; left: T = 4.98, p<0.001), and reduced it in the cerebellum, brainstem and periaqueductal gray matter (T = 11.52, p<0.001) (Fig 2, Table 1).

**Table 1:** Changes in brain metabolic activity during DBS in the three brain targets.

Target	ROI	Side	↑/ ↓	T	d	p value
**mPFC**	St, AA, Temp & Pir C	R	↑	6.39	2.13	< 0.001
	St, AA, Temp & Pir C	L	↑	4.98	1.66	< 0.001
	Hipp v	L	↓	7.07	2.57	< 0.001
	Cb, BS & PAG	R & L	↓	11.52	3.84	< 0.001
**NAcc**	Sub	L	↑	13.02	4.60	< 0.001
	Pir C	L	↑	6.52	2.31	< 0.001
	Pir C	R	↑	4.29	1.52	0.001
	Olfactory bulb	R & L	↑	5.20	1.83	< 0.001
	BS & PAG	R & L	↓	4.82	1.70	< 0.001
	Hypoth		↓	3.25	1.15	0.005
	Septum		↓	5.27	1.86	< 0.001
**DM**	St, NAcc & Pir C	R	↑	7.25	2.30	< 0.001
	St, NAcc & Pir C	L	↑	3.73	1.18	0.002
	Th	R & L	↑	7.78	2.46	<0.001
	Temp C	R	↓	3.43	1.10	0.003
	Temp C	L	↓	4.58	1.45	0.001
	Cing C	R & L	↓	3.64	1.15	0.002

Brain metabolic changes according to the stimulated target. Region of interest (ROI), side (left and right), glucose metabolism (increase: ↑ or decrease: ↓) t value (T), d Cohen (d) and statistical p value (p). AA: amygdala; BS: brainstem, Cb: cerebellum, CC: cingulate cortex, Hypoth: hypothalamus, NAcc: nucleus accumbens, PAG: periaqueductal gray matter, Pir C: piriform cortex, Sub: subiculum hippocampal, Str: striatum, Temp C: temporal cortex, Th: thalamus].

NAcc-DBS treatment increased metabolic activity in the left subiculum (T = 13.02, p<0.001), piriform cortex (right: T = 4.29, p = 0.001; left: T = 6.52, p<0.001) and olfactory bulb (T = 5.20, p<0.001), and decreased ^18^FDG-uptake in the brainstem and PAG (T = 4.82, p = 0.001), septum (T = 5.27, p<0.001) and hypothalamus (T = 3.25, p = 0.005) (Fig 2, Table 1).

DM-DBS treatment increased metabolic activity in the striatum, NAcc and piriform cortex (right: T = 7.25, p<0.001; left: T = 3.73, p = 0.002) and thalamus (T = 7.78, p<0.001) and decreased ^18^FDG-uptake in the temporal (right: T = 3.43, p = 0.003; left: T = 4.58, p = 0.001) and cingulate cortex (T = 3.64, p = 0.001) (Fig 2, Table 1).

## Discussion

To the best of our knowledge, this is the first comparative report on the use of small animal ^18^FDG-PET and SPM techniques in rats in an attempt to identify and compare the modulation of brain metabolic networks by DBS in the mPFC, NAcc and DM. We show that the effects of high frequency DBS on neuronal activity, reflected as the differences in regional glucose metabolism between DBS on and off conditions, involve modifications of complex networks rather than global or isolated regions. This is in agreement with our previous study for mPFC and NAcc stimulation in an animal model of schizophrenia [12]. Its capability to either increase or decrease activity supports the notion that DBS induces several mechanisms that lead to net inhibitory and excitatory effects irrespective of the function [48], suggesting a complex modulation of activity along cortico-basal ganglia-thalamo-cortical and the cerebello-thalamo-cortical circuits. Overall, stimulation in each brain target influenced a different set of structures at a distance from the target that might be relevant for addressing specific pathological conditions.

### Common DBS effects across different targets

DBS to all three targets induced increased metabolic activity in the piriform cortex (PC). The PC is the largest area of the mammalian olfactory cortex, receives direct projections from the olfactory bulb and contains the most susceptible neural circuits of all forebrain regions for electrical (or chemical) stimulation [49, 50]. Thus, immunohistochemical studies have shown that during electrical stimulation of limbic brain regions, the PC exhibits the most consistent increase in glucose utilization [49], similar to our results.

Another interesting finding is that both mPFC-DBS and NAcc-DBS decreased glucose metabolism in the brainstem. The mPFC is reciprocally connected with the dorsal raphe nucleus, which contains most ascending serotonergic neurons, and the ventral tegmental area (VTA) which contains mesocortical dopaminergic (DA) neurons, which could account for the decreased glucose metabolism seen in the brainstem. The medium spiny neurons of NAcc receive input from both dopaminergic neurons in the VTA and the glutamatergic neurons of the hippocampus, amygdala and mPFC. Thus, stimulation of NAcc at high frequencies could lead to an inhibition of dopaminergic activity at the brainstem level, resulting in decreased glucose metabolism in the brainstem. Our results are in line with that reported with citalopram, an antidepressant medication, showing decreased blood oxygenation level dependent (BOLD) signal in the brainstem using pharmacological magnetic resonance imaging [51]. In this sense, both brain targets have been recently proposed as targets for DBS in resistant major depressive disorder [52, 53], and has been associated with antidepressant, anxiolytic, and precognitive properties.

### mPFC-DBS increased brain metabolism in the temporal cortices

Hypofrontality is related to deficits in attention, memory and executive function, apathy, social withdrawal, restricted affection or anhedonia [54]. It has been suggested that the direct stimulation of the PFC may serve to modulate temporo-parietal attentional networks involved in the automatic processing of salient stimuli [30], playing a critical role in mood regulation [55]. In this sense, cortical stimulation for treatment-resistant depression constitutes a brain stimulation approach that has shown promise [56–58]. Here, we show that mPFC-DBS affected metabolic activity in the striatum, temporal and piriform cortices, the amygdala, cerebellum, brainstem and periaqueductal gray matter. This is in line with the PFC projecting to the ventral striatum and the head of the caudate, as well as other subcortical connections, including the amygdala [59]. Thus, our results showing an increased metabolism in temporal cortices support the notion that stimulation of mPFC could be explored for improving the attentional network. Moreover, behavioral experiments should be performed to corroborate these findings.

Cerebellar affectation has been commonly reported in schizophrenia, autism, and other developmental disorders [60–62]. Recent neuroanatomical evidence has also demonstrated closed-loop connectivity between prefrontal cortex and the cerebellum [63]. Moreover, electrophysiological and anatomical studies have demonstrated the existence of a prefrontal-olivo-cerebellar pathway in anesthetized mice [60], and the existence of disynaptic fronto-cerebellar connectivity in rats [64]. Our data showing that mPFC-DBS decreased glucose metabolism in the cerebellum, confirm the existence of a rodent prefrontal-cerebellar network [65, 66].

### NAcc-DBS increased brain metabolism in the subiculum

The NAcc has traditionally been associated with reward, pleasure and addiction, behavioral categories/systems implicated in the pathophysiology of basically all psychiatric disorders [16, 67–69]. In fact, the ventral capsule/ventral striatum (VC/VS), which includes the NAcc, is the unique brain target with FDA approval for DBS treatment of a psychiatric condition (OCD). The NAcc receives major dopaminergic afferents from mesolimbic origin, and dopamine is the most important transmitter within these nuclei. Thus, NAcc stimulation may lead to direct interferences in the dopaminergic system, or possibly indirect influences on the synaptic efficiency of this neurotransmitter system, with a huge spread of metabolic changes in the brain. Given its vast pathophysiological implication, network effects of NAcc-DBS were less striking and limited to the subiculum, piriform cortex (PC), olfactory bulb (OB), and brainstem. Off note, findings basically correspond to NAcc-DBS we recently reported using a functional MRI approach [70]. Of those effects, the increase of glucose metabolism in the subiculum is of particular interest. Neuroimaging and neuropsychological studies have shown an hippocampal dysfunction in Alzheimer's disease, cognitive ageing, post-traumatic stress disorder, obesity, schizophrenia, and depressive and anxiety disorders, among others [71]. Specifically in schizophrenia, there is robust evidence of hippocampal dysfunction, with impaired activation during memory tasks, increased baseline hippocampal perfusion, and reduced dentate gyrus neurogenesis and efferent signaling [72]. Moreover, obesity has been associated with defective hippocampal activity, which leads to cognitive deficiency in obese patients [73]. In this context and according to our results, it seems reasonable to explore the idea of applying NAcc-DBS in pathologies associated with hippocampal dysfunction.

### DM-DBS increased brain metabolism in the thalamus

The dorsomedial thalamus (DM) has strong interconnections with the dorsolateral PFC and limbic structures, besides being a critical element in the attentional “selective engagement” system. The dysfunction of this “sensory gating apparatus” has been associated to hallucinations, a common symptom in psychosis [74, 75]. At present, DM-DBS has only been applied experimentally in animal models [21, 53, 76–79]. Here, we found that DM-DBS affected metabolic activity in the striatum, NAcc, piriform cortex, medial thalamus and temporal and cingulate cortices. This is in line with the DM projections to the dorsolateral prefrontal and orbitofrontal cortical areas, which together project to the anterior cingulate cortex [80] and to the dorsal and ventral striatum [81]. Among those effects, the increase of glucose metabolism in the thalamus is especially relevant from a translational point of view. Neuroimaging has shown abnormalities in the DM of schizophrenic patients, with decreases in the thalamic D2 receptor binding [82], less prominent thalamic glucose metabolism rate [83], decrease functional connectivity of DM to other circuit areas or decreases in the thalamic blood flow [84]. Patients with frontotemporal lobe degeneration associated with dementia also shown decreased glucose metabolism in the medial temporal region, the thalamus and striatum [85]. In Alzheimer disease, thalamic abnormalities at the anterior thalamic nuclei have been associated with cognitive deficits in memory and attention [86]. In view of these studies and our results, it seems essential to explore the idea of applying DM-DBS in pathologies associated with cognitive deficits in memory and attention and dementias.

### Limitations of the study

Our study is subject to two limiting factors. The first is the use of naïve animals to study DBS’ effects. Clearly, in the clinic, DBS is applied to diseased brains and its therapeutic effects are a function of its interaction with altered brain network. Another limitation is related to the temporal influence of DBS as studied here; we used an acute stimulation protocol preceding the PET scans acquisition. In the clinical scenario, DBS is applied chronically and usually therapeutic effects evolve over a timeline of stimulation.

## Conclusion

In conclusion, we show that DBS in mPFC, NAcc and DM induced different patterns of ^18^FDG uptake despite sharing interconnections with the same circuitry, and this may have important implications to DBS research suggesting individualized target selection according to specific neural modulatory requirements.
